# Issues in evaluation of cognition in the elderly in developing countries

**DOI:** 10.4103/0972-2327.41874

**Published:** 2008

**Authors:** R. Mathew, P. S. Mathuranath

**Affiliations:** Department of Neurology, Cognition and Behavioural Neurology Center (CBNC), Sree Chitra Tirunal Institute for Medical Sciences and Technology (SCTIMST), Trivandrum - 695 011, India

**Keywords:** Cognition, dementia, developing countries, elderly, impairment, scale, screening

## Abstract

**Background:**

Developing regions of the world host the majority of elderly subjects who are at risk for dementia. Reliable epidemiological data from these countries is invaluable in tackling this global problem. Scarcity of such data in literature is largely attributable to problems that are unique to developing communities worldwide.

**Objective:**

To classify and describe the problems that interfere with the collection of reliable epidemiological data on cognitive impairment in the elderly in developing communities, and to suggest practical solutions for some of them.

**Methods:**

Inferring from the experiences of a large, ongoing, population-based study on the cognitive impairments in the elderly in South India and from the review of literature.

**Conclusion:**

A fatalistic attitude regarding aging in the communities, significant heterogeneity in educational abilities and activities of daily living, high illiteracy among rural subjects, and lack of an organized health care system and updated demographic figures are some of the major factors that contribute to technical, namely, methodology-related problems and practical, namely, subject-related problems in such epidemiological studies.

## Introduction

Considerable epidemiological data available on dementia come from the developed world. Over the past two decades, however, it is being increasingly recognized that a large number of patients with dementia live in the developing world; this accounts for more than 50% of the global elderly population, and India and China contribute to more than half of this number.[1] In addition to the large populations of these two countries, increasing average life expectancy of individuals at birth, a byproduct of the economic progress made by both these countries over the past two decades also contributes to it. Very few population-based studies have been conducted on the prevalence or the types of dementia from these two countries[2–6] or from other countries in the developing world.[7,8] Clearly, there is an urgent need for accumulation of more epidemiological data on this subject from the developing world. Problems that are associated with the execution of such studies and that are unique to developing countries constitute an important reason for the dearth of these data. Successful planning and execution of future epidemiological studies on dementia in the developing countries will benefit from a prior knowledge of these problems.

A community-based epidemiological survey for the prevalence of cognitive impairment in the elderly (>60 years) individuals is underway in the city of Trivandrum, in the state of Kerala, South India. The literacy rate of this city is 81%, with literacy defined as an ability to read and write in one's native language.[9] The study is being conducted in two phases, Phase I for screening and Phase II for detailed clinical and neuropsychological evaluation and confirmation of diagnosis of dementia. Phase I of this survey consisted of a door-to-door screening of all subjects 60 years of age or more. The screening instruments include a demographic and risk-factor questionnaire, a global cognitive screening instrument, Addenbrooke's Cognitive Examination (ACE), which includes the Mini-Mental Status Examination (MMSE) and an Instrumental Activities of Daily Living (IADL) Scale. The latter two instruments have been culturally modified and adapted for the local population and norms (education-specific for ACE and MMSE) have been derived. A random cluster sampling technique was used to select the target population. The target population consisted of 2,932 elderly individuals from a population of 41,920, which in turn, constitutes approximately 8% of the total population (524,006) of Trivandrum City. From the target sample, 2,467 subjects completed Phase I, while the remaining 465 (15.8%) did not. All individuals scoring below the 20^th^ percentile on the global cognitive screening test and a random selection of 10% of those scoring above this percentile are being evaluated in Phase II.

Inferring from the experiences of this ongoing population-based study and after a review of English literature for problems reported by other investigators in similar settings, this article formulates the issues related to cognitive screening of the elderly in developing countries and briefly discusses effective solutions for some of them.

Any study on dementia and related disorders focuses on the elderly in the population since it is primarily a disease of the elderly. The acceptance of ill health as an unavoidable burden of old age and a fatalistic attitude regarding aging in many communities in the developing world makes the elderly and their families reluctant to seek medical help. The present cohort of the elderly in most developing countries is very heterogeneous with respect to educational level. A substantial proportion is illiterate and predominantly from a rural background. These factors make it less likely that the cognitive impairment in them will be recognized or help will be sought sufficiently early. Understanding these characteristics and the problems arising thereby is necessary for any successful planning and execution of such studies. Broadly, these problems or issues can be categorized into two practical and technical issues [Table 1]. Technical issues pertain to those problems that are associated with the design of the study and are mainly dependent on the methodology adapted. The investigators can partially (or completely) eliminate the biases arising thereof, by taking into account these issues while designing the study. Practical issues pertain to the problems that relate to the population being surveyed (characteristics and behavior) and are prevalent in the field. These issues are often beyond the control of the investigators, and can seldom be eliminated completely. Knowledge of practical issues, however, helps in planning the study in a manner in order to minimize the biases introduced by them. It is important to understand that both factors have an interaction and cannot be addressed individually. In this article, we first address practical issues since this includes the description of the unique characteristics of the population, the knowledge of which is of immense importance in understanding the technical issues.

**Table 1:** Classification of issues related to epidemiological surveys for cognitive status of the elderly in developing countries.

I.	Technical Issues
	Selection and training of personnel
	Environment of testing
	Test battery
	Measures of activities of daily living
	Sampling and statistical methods
II.	Practical Issues
	Demography
	Motivation and cooperation
	Informed consent
	Reliable informant and accuracy of information
	Comorbid conditions in target population
	Follow-up

## Practical Issues

### Demography

In developing countries, while the majority of the population lives in rural areas, a pattern of migration is noted (either permanent or temporary), with a constant influx into the local urban population and out flux from the rural population. Similarly, one of the problems encountered in the ongoing study in South India was that a proportion of the elderly people surveyed had permanent residence in a different region; however, they were presently staying on a temporary or intermittent basis for a variable duration of time (months to years), in the house of a relative, usually an offspring. In order to generate significant estimates or comparisons from any epidemiological data, it is necessary that the sample be representative of the population from which they come and to which the results will be generalized. A stable population, with a low rate of migration in and out of the community, therefore, is an essential requirement. One method of mitigating the problem related to migration is to select a survey site that includes an urban region and its surrounding rural region, which constitutes a potential source for such migrations.

Another important factor influencing the selection of the survey site is the accessibility of the proposed site and of the individual units within the proposed site.[10] In some of the developing countries, these factors may not always be favorable if the target population includes subjects living in interior rural areas.

### Motivation and cooperation

In economically backward communities, we encountered resistance from the relatives of some of the subjects, especially where the subject was financially dependent on the relatives. In most such cases, this was because of the misconception that the detection of disease would mean prolonged treatment, leading to considerable financial burden or because there were no immediate financial gains from such a participation. Some families believed that deterioration in the health of the elderly was a part of the aging process, and therefore, did not require any concern. Consequently, these families were not keen to expend resources on the health of the elderly. Jamuna *et al* in 1990 clearly illustrated these issues and found that many caregivers felt that caring for the elderly was stressful, taxing, unrewarding and resulted in a financial burden for the family.[11]

Lack of awareness among potential participants and their families regarding the health of their elderly and the utility of such surveys, often leads to low participation rates and poor cooperation. Creating awareness regarding these issues, therefore, should precede the survey and is best achieved through local sources of information. As we realized, such a strategy helps in priming potential participants and their relatives for the survey. Awareness can be generated in the form of simple articles or advertisements in local newspaper(s), posters at primary health centers and other public places and periodic announcements made by local health workers and community leaders at local gatherings or meetings, e.g., places of worship, local councils, etc. Creating awareness should also highlight the economic gains associated with early detection of diseases and initiation of proper management, such as prevention of problems, e.g., wandering, falls, fractures and secondary medical problems due to poor self-care, all of which can result in economic loss. It should also emphasize that estimating the burden of the disease in the community is invaluable for introducing management strategies such as group counseling and caregiver's support groups. Conducting free medical camps also helps in creating awareness and generating the confidence of the community. However, during our study, we felt that such camps are more beneficial to the community if they follow, rather than precede, the survey.

Some investigators suggest that the cooperation of the local physicians and traditional healers who often have a large following amongst the local population, especially in rural areas, is also an important factor.

Studies on factors affecting participation rates have shown that low levels of literacy, poor physical health and impaired cognitive function were negative factors associated with willingness in the elderly people to participate in such surveys.[12] The first of these factors is significant in the developing countries, especially in rural areas and more so in the aging population. Interest in the project, realizing the potential benefits and having a positive attitude toward research were the positive factors associated with the willingness to participate in such surveys.[12]

### Informed consent

Many investigators have emphasized the difficulty in obtaining informed consent from study participants or their relatives, especially in the rural areas with low literacy levels.[13] It is useful if the consent form is brief, in the local language and avoids scientific terms. It is helpful to have local health workers and leaders from within the community to explain to the participants the purpose of, the need for, and the obligations related to the consent. Although we did not experience this, we are aware that some participants, especially illiterate, are unwilling or incapable of signing written consent, while they are happy to give verbal consent. In such situations, recording the verbal consent in the presence of a neutral witness can circumvent the difficulty associated with written documentation.

### Reliable informant and accuracy of information

In some developing parts of the world, acquiring reliable and accurate information is often difficult in rural areas. The informants, generally the relatives of the subject, are often employed and may not be available to provide the necessary details about the subject if the house-visits are conducted in the daytime. House visits on holidays or in the evenings are likely to be more productive.

In India, as in most developing countries, the elderly often live along with the families of their offspring(s), especially in rural areas. In case of more than one offspring, they tend to move from the house of one offspring to that of another, with variable periods of stay with each family. In such individuals, continuity in the history requires multiple informants, which may not be easily available. Vital information relating to date of birth, education, previous illness, present medical illness, and present medications may not be available in many of the situations. Accurate age and date of birth are often not maintained in rural areas. In such situations, an estimate of the year of birth can be made by relating it to event(s) that may have taken place at a local or national level during that time or by relating it to the ages of the children of these subjects. As the incidence and prevalence of dementia increases exponentially with age, lack of information on age affects reporting of age-specific rates and ratios.[14] Another unique problem that we encountered, especially among elderly females, was that some subjects who had no formal conventional education were capable of reading and writing. They were either self-taught or had received instructions in the local system of education that was popular in the community (e.g., ‘Madrasa’ in the Muslim communities). In contrast, some subjects who claimed to have received formal education (sometimes up to three years) were completely incapable of any reading or writing. These details should be properly explored and the level of literacy should be estimated by the educational abilities of the subject rather than by the number of years of formal education. The method(s) of estimating educational ability by using tasks such as reading the headlines of a newspaper; writing a letter, a sentence or one's address, etc., should be derived and standardized in advance.

### Comorbid conditions in the target population

Age-related nonneurological problems often interfere with proper cognitive screening. An epidemiological survey on Indian elderly reported that hypertension, diabetes, joint pain, cataract, ulcers, limb paralysis, gastrointestinal malignancies, etc., are highly prevalent diseases of the elderly.[11] Problems such as cataract and presbyacusis, which frequently remain uncorrected in the rural elderly, directly interfere with their performance on cognitive screening tests. A general medical camp for detecting these problems and advising corrective measures such as undergoing cataract surgery or using hearing aids, although helpful, is not always cost effective. An alternative is to use test materials or methods that will be compatible with these patients (discussed later in the article). In addition, a neurologist should examine these subjects and exclude any cognitive impairment. Another frequently encountered problem, which can interfere with the cognitive screening, is exposure to medications (indigenous or otherwise) the names or pharmacological properties of which may be unavailable to the investigators. Even if they are available, the influence of indigenous medication on the subject's cognitive abilities may be unknown. At the time of priming the community, we found it helpful to request all subjects to consult their doctors and temporarily stop all nonessential medications and restart it after the survey was over. Priming should precede the house visits by two to three weeks in order to permit a washout period for such drugs.

### Follow-up

It is mandatory that patients who are identified with neurological disorders in the survey receive appropriate long-term treatment, and if required, additional diagnostic procedures be performed at the nearest medical facility.[10] Follow-up, therefore, is essential in such patients and in those who are a part of a longitudinal study. Lack of locally available medical facility, especially in rural areas, makes it difficult for the patients to receive long-term treatment. Unfortunately in many developing countries, the existing policies of the state do not allocate resources toward such activities. This, in turn, serves as a disincentive for other members of the community for participating in any longitudinal study. Consequently, there is an added responsibility on the sponsors of such studies to provide opportunity to patients to undergo the necessary follow-up by organizing medical camp(s). Even with limited resources, we found this to be an effective means of gaining the confidence of the community. This helps in improving their participation in longitudinal studies. It is imperative that during the planning stages of such studies, especially if they are longitudinal, resources be allocated to these aspects of the study since they are invaluable in improving the quality of the study.

## Technical Issues

### Selection and training of personnel for administering the screening battery

In developing countries, few qualified neuropsychologists are found. Hence, lay workers have to be specially trained to perform the fieldwork of administering the interview schedule and the cognitive screening batteries. As far as possible, these personnel should be selected from within the target population and should be sharing its culture. It is not uncommon to find such field workers maintaining their distances from the investigators/scientists. It should be ensured that the field workers feel that they have access to the scientists/investigators and do not feel intimidated.[13] It is also necessary to reduce reliance on independent judgment of these workers as far as possible during the interview. For this effect, the rules for administration of the test batteries should be made as explicit as possible, and standard protocols should be provided for dealing with all foreseeable situations that may arise during testing.

### Environment for conducting the test

The patient's attention is very important in order to assess them with screening tests. Any source of distraction can confound the results, especially in subjects with low cognitive reserves. In most developing countries, it is not surprising to find a number of adults and children cohabiting with the subject. In urban areas, the limited living space results in high population density. In such circumstances, it is nearly impossible to attain the optimal testing conditions for the screening tests. It is important, therefore, to consider these factors before interpreting the test results. Most subjects have significant pretest anxiety. Elderly illiterate subjects, especially in rural areas, are also easily perturbed by novel experience and feel intimidated by sophisticated urbanites. It is no surprise that their performance on the screening instruments is often not their best one. The investigators should anticipate such circumstances in advance since these factors constitute avoidable or modifiable confounders. It is helpful, therefore, to recruit local residents and train them as field workers. They should also be trained to develop a good rapport with the subject before administering the actual test. Provision should be made at the planning stages of the study for all these factors. It has been our experience and also that of others[13] that some subjects are often helped by a demonstration of the testing procedure on dummy subjects. Dummy tests or test items that are comparable in their complexity with the original tests, graded in difficulty, educationally fair, having a high probability of success and unlikely to produce a practice effect on the onlooker, should be used in such situations. If used at all, they should be used on all subjects to maintain uniformity of the testing procedure. It is worth emphasizing that field workers recruited and trained from the local community are more effective in carrying out the interview and administering the screening instruments since the subjects are familiar with them. Interviewers should take every care to keep the patient comfortable and relaxed, yet retain sufficient authority to create the necessary seriousness and involvement with the subject.

For testing the illiterate subjects who have never been exposed to test situations, the respondents will frequently request clarifications of the instructions. All the responses have to be recorded verbatim. A taped recording of the responses enables the neurologist to analyze the responses better, and if required, modify the test battery. Cognitive testing in any population requires that the subject's best possible responses be obtained in a uniform and standardized manner. This may not be very easy in an illiterate population. In many situations, the respondents may not be very clear as to what is exactly expected of them. In such situations, tests may have to be repeated. These situations have to be foreseen and the tests, which can be repeated, and the number of times to repeat them should be formalized in advance.

### Test battery

In most population-based surveys, a subject-based neuropsychological screening instrument is used. These are generally one of the global cognitive screening instruments such as the MMSE[15] and Cognitive section (CAMCOG) of the Cambridge Examination for Mental Disorders of the Elderly (CAMDEX)[16,17]. These instruments should first be adapted for the local population by making it linguistically and culturally fair for the population being studied. It is also important to ensure that the modified components of the instruments match in complexity with the original item and are not influenced by factors other than those that influence the original item. The importance of this is well-illustrated by the study by Ratcliff *et al*,[18] who attempted to replace the letter fluency task with phonemic fluency task in order to adapt it for an illiterate population and found that the latter was indeed influenced by the knowledge of written language, and therefore, was not of use in the illiterate population.

In almost all the global cognitive screening instruments, education level of the subject significantly influences the performance in the test.[19–23] It is, therefore, necessary that education-specific cut-off scores be used in arriving at the final results to avoid spuriously increased rates of dementia prevalence among the low education group.[24] It is also important that these cut-off scores be derived on either the study population itself or on a population similar to it with regard to physiognomy since the education-specific cut-off scores also vary with the characteristics of the population. Katzman has also suggested that education-specific cut-off scores on mental status examination are useful for selecting subjects for more intensive clinical evaluations.[25]

Ideally, the tests should not have significant flooring or ceiling effects, and the distribution of scores should be comparable to that observed in other similar populations. During the stages of adaptation and validation of the screening instrument used by us, we found that even among normal (control) subjects, a marked difference existed in the performance in the same version of the test between illiterate and literate populations. This often resulted in flooring effects (for the illiterate groups) on some of the test items. These effects were difficult to eliminate despite modifications in the grades of difficulty or the scoring system. This problem is likely to arise if the survey population is very heterogeneous in terms of levels of education, as was the case with our sample. Using education-specific cut-off was helpful in reducing this problem to some extent. Equally complex is the problem of ceiling effects. We realized that certain items in a test could not be attempted by some sections of the population. For example, using complex two-dimensional figures (such as wire cube) for construction in testing-produced flooring effect in the illiterates who had difficulty in writing but produced ceiling effect among the literates (thereby making it less sensitive in early detection of impairment in them). Eliminating such items from the battery could result in substantially reducing the scope of the battery for comprehensive cognitive assessment. In retrospect, we feel that in such situations, future studies should investigate the possibility of using different versions of the tests, modified appropriately for the population being tested. It is essential to ensure that different versions have comparable sensitivity. It is important that the concurrent validity of neuropsychological tests on the study population in terms of specificity, sensitivity and predictive values are established by a pilot study preceding the survey. In our experience, the subject's performance on the screening test is significantly affected by the limitations imposed by physical handicaps such as impaired vision or hearing. Using appropriately large print size in the test materials, including the size of pictures, could partly remedy this problem. Subjects with impaired hearing often benefited from written instructions, which were prepared in advance. We also found that a slow and animated demonstration of dummy tests on dummy subjects was also helpful in circumventing this problem to some extent. Nevertheless, these problems cannot be completely eliminated, and therefore, it is important to state in the final report, the biases, if any, introduced by these factors.

### Measurement of activities of daily living

Very few measures of activities of daily living (ADL) that are appropriate for the elderly living in developing countries are currently available. In the urban areas, more and more elderly are tending to be living on their own and carry out all ADL themselves. In contrast, the needs of most elderly living in the rural areas are taken care of by the younger members in the joint families. Few, if any, of the conventional activities, such as holding a formal employment, handling finances, are expected of them. Consequently, very little demand is placed on cognition in carrying out their ADL. A substantial compromise in cognitive reserve, therefore, is necessary before it can impair their ADL. Thus, a discrepancy between the scores obtained by cognitive screening instruments and functional scales is not uncommon. In such circumstances, the variations in the prevalence rates of dementia, attributable to the definition of dementia used, becomes grossly exaggerated.

Moreover, a considerable gender difference also occurs in the activities performed. For example, cooking, is seldom performed by men, and managing home finances, seldom by women. Thus, the residential area (i.e., rural or urban), as well as the gender, result in considerable heterogeneity in the ADL. In developing test items, therefore, in addition to professionals, leaders and health workers from the local community should also be consulted,[26] and items should be weighted for factors such as gender and residential area. In addition to being easy to administer and score, each item (assessing a particular task) should elicit multiple responses that are objectively graded in terms of difficulty in performing the task. In situations where the majority of the population is illiterate, this gradation may have to be sometimes limited to a two-point scale (e.g., can or cannot perform the activity) in case a higher point scale tends to elicit an ambiguous response or confuses the responder. In view of the significant physical disability that is present in many of these subjects, it is important to ensure that the given response is the result of cognitive and not physical impairment. The methods used for obtaining the information (e.g., alternative phrasing, probing, repetition) should be recorded since it may help in further refining the instrument. As with the cognitive screening instruments, the ADL instruments should also be validated by using on the population in question and norms derived from the same.

### Sampling and statistical methods

For an epidemiological study on prevalence, a stratified random sample is preferred over a cluster random sample. Stratified random sample requires prior knowledge of the basic demographic structure of the entire population. In densely populated rural areas in developing countries, this information may not be always available to the investigators, or if available, may not be a recent data. However, rural areas that are close to urban medical schools often have well-defined regions that are cultivated over many years by the community medicine departments of these medical schools as field practice areas. The population demographies of such regions are often available and frequently updated. For rural surveys, if available, such sites are preferable since they provide a stratified random sample. This is one of the reasons why some of the neuroepidemiological studies such as the one conducted in Nigeria,[7] North India[2] and South India[27] selected communities where local medical schools or hospitals have traditionally held out-reach centers. In most other situations, one has to settle for a cluster random sampling technique.

In developing regions, the quantum and the significance of sample attrition are very difficult to predict at the planning stages of any epidemiological study. In the study by Froukje *et al*,[28] of the 17.5% nonresponders, 13.9% were due to refusal. Compared to responders, nonresponders were less often institutionalized. Considering all the above factors, the rate of nonresponders is likely to be higher in the developing world, especially in rural areas. It is important, therefore, that the study design incorporate procedures to sociodemographically describe a nonresponse and in relation to the study objectives. Similarly, in order to improve the validity, the design should incorporate categorization of the nonresponders into distinct groups.

Another observation in our study, and so also in other studies from developing countries,[2] is that women are frequently noted to be preponderant in such surveys. This may be associated with gender differences in health service utilization, or to the fact that they are confined to their homes due to their roles as housewives, and therefore, more easily available for interview than men. Such a gender bias is of profound significance in surveys on cognitive health of the population. A large proportion of women continue to remain in their occupational roles as housewives beyond the retirement age long after many men of similar age have reduced their sphere of activity in the work place to less demanding ones in the home and community. Thus, dementia-related deficits in social and occupational functioning might be detected earlier in elderly women than in elderly men. Only repeated examination of men and women with the same objective measures can uniformly detect impairment and disease in the individuals.

### Recommendations

Investigators performing epidemiological studies on cognition (and evaluating cognition) in countries such as India should specially focus on the methodology to improve the accuracy, reliability and validity of their results. Potential investigators should recognize that such studies are the results of prolonged well-planned efforts of a multidisciplinary team. Approval for epidemiological studies from central or institutional ethics committees and procedures for obtaining informed consenting from subjects should constitute an integral and fundamental part of such studies. It is important for investigators and team members to understand and learn the methodology of tool development. Tools for global cognitive screening and for measuring ADL that are culturally and linguistically fair for the target population should be diligently developed, validated and standardized not arbitrarily but systematically by following the methods prescribed for such procedures. Subsequently, education-stratified norms on these procedures should be derived for the local population. It is also important to train the field staff for adequate length of time and periodically supervise, inspect and cross-examine their work in order to bring homogeneity in the evaluation process. This training should also include the assessment of the competence of hearing and vision in subjects for performing the neuropsychological evaluation. At the time of data collection and analysis, investigators should systematically include all types and causes of attrition to avoid under- or overestimation of the problem. Caution and care exercised on these issues by investigators from the beginning of the planning of the study will enhance the reliability and accuracy of the results of the study.
